# Avian Influenza Infection Alters Fecal Odor in Mallards

**DOI:** 10.1371/journal.pone.0075411

**Published:** 2013-10-16

**Authors:** Bruce A. Kimball, Kunio Yamazaki, Dennis Kohler, Richard A. Bowen, Jack P. Muth, Maryanne Opiekun, Gary K. Beauchamp

**Affiliations:** 1 United States Department of Agriculture, Animal and Plant Health Inspection Service, Wildlife Services, National Wildlife Research Center, Philadelphia, Pennsylvania, United States of America; 2 Monell Chemical Senses Center, Philadelphia, Pennsylvania, United States of America; 3 United States Department of Agriculture, Animal and Plant Health Inspection Service, Wildlife Services, National Wildlife Research Center, Fort Collins, Colorado, United States of America; 4 Department of Biomedical Sciences, Colorado State University, Fort Collins, Colorado, United States of America; 5 Department of Microbiology, Immunology and Pathology, Colorado State University, Fort Collins, Colorado, United States of America; NIH, United States of America

## Abstract

Changes in body odor are known to be a consequence of many diseases. Much of the published work on disease-related and body odor changes has involved parasites and certain cancers. Much less studied have been viral diseases, possibly due to an absence of good animal model systems. Here we studied possible alteration of fecal odors in animals infected with avian influenza viruses (AIV). In a behavioral study, inbred C57BL/6 mice were trained in a standard Y-maze to discriminate odors emanating from feces collected from mallard ducks (*Anas platyrhynchos*) infected with low-pathogenic avian influenza virus compared to fecal odors from non-infected controls. Mice could discriminate odors from non-infected compared to infected individual ducks on the basis of fecal odors when feces from post-infection periods were paired with feces from pre-infection periods. Prompted by this indication of odor change, fecal samples were subjected to dynamic headspace and solvent extraction analyses employing gas chromatography/mass spectrometry to identify chemical markers indicative of AIV infection. Chemical analyses indicated that AIV infection was associated with a marked increase of acetoin (3-hydroxy-2-butanone) in feces. These experiments demonstrate that information regarding viral infection exists via volatile metabolites present in feces. Further, they suggest that odor changes following virus infection could play a role in regulating behavior of conspecifics exposed to infected individuals.

## Introduction

It has long been speculated that infections may cause odor changes in animals and humans [1]–[4]. For example, it was shown recently that infection with *Mycobacterium tuberculosis* produces unique volatiles in the breath of infected donors [5]. Several cancers have been detected from odors in breath or urine by trained dogs [6]. Much of the research involving volatile metabolites associated with infection has focused on parasites [7]–[9], though influenza [10] and mammary tumor virus [11] have been studied in mice. The mechanisms underlying alteration of body odor by infection are poorly understood, but they may involve alteration of immune function [11]. Odor changes may also result directly from disease pathology [12].

There is also convincing evidence that healthy individuals modify their social behaviors when exposed to infected conspecifics themselves or their body odors. Avoidance of individuals on the basis of odors associated with illness may serve to reduce the probability of disease spread [13]. Alternatively, it would be adaptive for the infective agent to increase both inter- and intraspecific interactions via attraction to these same odors. Attraction has been clearly demonstrated for interspecific interactions between human hosts and insect vectors [14].

The hypothesis that immune activation may produce a meaningful alteration of body odor led us to study odor changes resulting from administration of vaccines in mice (unpublished data). Using biosensor panels of trained mice, we demonstrated that body odor was altered by immunization with either rabies or West Nile Virus vaccine. Based on this promising result, we conducted similar experiments with an avian influenza virus (AIV) to evaluate whether infection produces a distinctive odor change in a relevant species; mallard ducks (*Anas platyrhynchos*). The mallard is considered an excellent research model for influenza research [15]. We examined feces because they are a source of social odors [16] and because they are the primary avian waste excretion product (unlike rodents which employ both feces and urine to serve this function). Signaling of viral infection could have important implications for social interactions in brood-forming species, such as mallards. Although auditory and visual senses are highly developed in avian species, the role of olfaction is often overlooked in birds. However, avian olfaction has been implicated in many behaviors such as navigation and foraging [17] and predator avoidance [18]. Thus, it is quite possible that infections could be recognized by brood mates on the basis of fecal odor change.

We hypothesized that infection with a low pathogenic influenza virus (H5N2) would alter the volatile profile of feces. We first postulated that these changes would be discriminable by mice trained in a Y-maze to identify odors associated with AIV infection. The Y-maze has successfully been used to discriminate between many different sources of odor variation, including: fetal odortype [19], [20], disease [11], [21], age [22], and diet [23]. Successful demonstration that infection resulted in fecal odor change verified that volatile emissions differ between the feces of AIV-infected and non-infected ducks. Having successfully demonstrated this occurrence, we employed a series of chemical analyses to identify volatiles that are significantly altered (qualitatively or quantitatively) in feces as a result of AIV infection.

## Materials and Methods

### Ethics Statement

This study was carried out in strict accordance with the recommendations in the Guide for the Care and Use of Laboratory Animals of the National Institutes of Health and the Guide for the Care and Use of Agricultural Animals in Agricultural Research and Teaching of the Federation of Animal Science Societies. Animal procedures were reviewed and approved by the Institutional Animal Care and Use Committees of the Monell Chemical Senses Center (#1123), USDA National Wildlife Research Center (#2021), and Colorado State University (#09-1317A).

### Duck Feces

Eight farm-raised mallards of mixed gender were used in this study. Six ducks were infected ocularly, intranasally and orally with 1 ml of brain heart infusion broth containing 1×10^6^ pfu/ml of A/Mallard/MN/346250/00 (H5N2). Cloacal swabs were collected on days three and four following experimental infection. Infectivity was confirmed by real-time RT-PCR and inoculation into 10-day old embryonating chicken eggs.

Two pooled feces samples were collected from each duck. Feces were collected daily for seven days immediately preceding experimental infection and again for days three through 10 post-infection. Eight pre-treatment and eight post-treatment samples were stored frozen at −80°C. To facilitate safe transport and handling of feces, all 16 samples were subjected to 2.7 Mrad of cesium irradiation for 27 hours. Lack of infectivity was confirmed by inoculation into 10-day old embryonating chicken eggs.

### Biosensor Assays

Six female C57BL/6 in-bred mice (born and raised in our laboratory) were trained to discriminate between feces from non-infected and infected ducks in a Y-maze as previously described [24]. The reward for correct response was a drop of water (the mouse having been restricted of water for 23 hours). Air was conducted through two odor chambers, each containing feces (0.5 g) exposed in Petri dishes for odor delivery to a maze arm. Gates placed at the entrance of each arm and at reward delivery locations inside the arms were manually raised and lowered in timed sequence to permit the training or testing of each mouse in a session of up to 48 consecutive trials.

In training sessions, fecal samples from three ducks (#3, 4, and 13; Table 1) were presented pair-wise (representing samples collected before and after experimental infection) and mice were rewarded for correctly choosing the maze arm associated with feces collected post-infection. For sessions 1 through 10, only one pre-infection and one post-infection sample were used (Table 2). Because the number of donors was limited, pairs (of samples with identical infection status) were used for sessions 11 through 13. Furthermore, pairs were frequently used for rewarded trials in generalization trials. Given the small number of donors, pairing of samples was used to artificially create unique odor stimuli. Training continued until each biosensor achieved greater than 80% concordance (correctly responding to feces from infected duck). Throughout training, about 25% of trials in a session were intentionally not rewarded, even if a correct response was evident.

**Table 1:** Donor IDs, treatments, and use of feces in behavioral assays.

Duck	Treatment	Biosensor Design
1	Control	Generalization
2	Control	Generalization
3	H5N2	Training
4	H5N2	Training
5	H5N2	Generalization
11	H5N2	Generalization
12	H5N2	Generalization
13	H5N2	Training

**Table 2:** Training session stimuli.

Sessions	Pre-treatment Donor	Post-Treatment Donor
1 thru 4	Duck 3	Duck 4
5 thru 7	Duck 3	Duck 13
8	Duck 3	Duck 3
9	Duck 4	Duck 4
10	Duck 13	Duck 13
11–13	Duck 3Duck 13	Duck 3Duck 13

Mice were rewarded for selection of the maze arm associated with feces collected after experimental AIV infection (post-treatment collections).

Sessions of unrewarded generalization trials were initiated when each biosensor demonstrated >80% concordance. Fecal samples from three infected ducks (#5, 11, and 12; Table 1) and two uninfected control ducks (#1 and 2; Table 1) were used in unrewarded generalization trials. These generalization samples were unfamiliar to the biosensors (not used in training trials) and were “double-blind” (the infection status of these paired samples was unknown to the maze operator). Each session consisted of four or five generalization trials interspersed among rewarded trials employing training samples. All within-duck comparisons (pre- versus post-infection) were subjected to the bioassay as well as some between-duck comparisons.

Cumulative responses of the full panel of trained mice were calculated for each generalization trial. Success rates (number of correct trials divided by the total number of generalization trials) were subjected to statistical tests of binomial proportion using the continuity correction for small numbers of observations [25]. A success rate of 0.5 was the null hypothesis for all tests.

### Fecal Analysis: Headspace Volatiles

Headspace analyses were conducted with a HT3 dynamic headspace analyzer (Teledyne Tekmar, Mason, OH, USA) outfitted with Supelco Trap K Vocarb 3000 thermal desorption trap (Sigma-Aldrich Co., St. Louis, MO, USA) attached to a Thermo Trace GC-MS equipped with a single quadrapole mass spectrometer (Thermo Scientific, Waltham, MA, USA) and a 30 m×0.25 mm id Stabiliwax-DA fused-silica capillary column (Restek, Bellefonte, PA, USA). Fecal samples (0.5 g) were maintained at 50°C, swept with helium for 60 min (flow rate of 75 mL/min), and the volatiles collected on the thermal desorption trap. Trap contents were desorbed at 260°C. The GC oven program had an initial temperature of 40°C (held for 3.0 min) followed by a ramp of 7.0°C/min to a final temperature of 230°C (held for 6.0 min). The MS was used in scan mode from 41 to 400 m/z.

Samples were analyzed in triplicate. Chromatographic data were converted to NetCDF format for baseline correction, noise elimination, and peak alignment processing using Metalign [26]. Multivariate results of this process consist of all mass spectrometric responses (m/z) exceeding a pre-defined threshold at each scan event. Sample means were exported to Unscrambler (Camo Software Inc., Woodbridge, NJ) for principal components analysis (PCA). An iterative process was used to select observations which contributed to a PCA model with visually evident segregation of AIV infection status in 2D scatter plots. Original chromatographic data were evaluated to determine the potential identity of the volatile compounds giving rise to the PCA results.

### Fecal Analysis: Solvent Extraction

Semi-quantitative analyses of acetoin (3-hydroxy-2-butanone) and 1-octen-3-ol in fecal samples were conducted by subjecting 0.25–0.75 g fecal samples to extraction with 10.0 mL ethanol (Fisher Scientific, Pittsburg, PA) in 25-mL screw-cap culture tubes. Analytical standards were obtained from Sigma-Aldrich (Milwaukee, WI). The tubes and contents were placed in a vortex mixer (Fisher Multitube Vortexer, Fisher Scientific) for 30 minutes and centrifuged at 2000 rpm (Thermo IEC Centra CL2, Thermo Scientific, Waltham, MA). Non-volatile extractives (e.g. plant pigments from duck diets) were removed by passing green-colored extracts through graphitized carbon solid phase extraction (SPE) cartridges (Supelco, Bellefonte, PA). Sample eluates were colorless, indicating that plant pigments were removed from the solvent extracts.

Splitless injections (1 µl) were made into a Thermo Scientific ISQ GC-MS equipped with a single quadrapole mass spectrometer (Thermo Scientific) and a 30 m×0.25 mm id Stabiliwax-DA fused-silica capillary column (Restek, Bellefonte, PA, USA). The GC oven program had an initial temperature of 40°C (held for 1.0 min) followed by a ramp of 3.0°C/min to 112°C and a ramp of 25°C/min to 235°C (held for 3.0 min). The MS was used in scan mode from 33 to 400 m/following an 8.5 min solvent delay. Selection ion monitoring (SIM) chromatograms were produced from the sum of the m/z responses of 45 and 57. Peak area responses were normalized by dividing peak area by sample mass (g). Peak area ratios were also calculated by dividing the 1-octen-3-ol peak area response by the acetoin response. Peak and peak ratio responses were evaluated by t-test to determine if they were impacted by AIV infection. Because the pre-treatment fecal sample from duck 11 was exhausted during bioassays, peak responses from extracts of the post-infection sample were not included in statistical analyses.

Several experiments were conducted to evaluate method performance. A standard solution containing both acetoin (5.69 µg/mL) and 1-octen-3-ol (5.05 g/mL) was repeatedly passed through SPE columns to evaluate irreversible loss of the analytes. Replicate extractions were also conducted with the post-infection sample from one duck (duck 5) to evaluate repeatability.

## Results

### Viral Infection

Results of real-time RT-PCR and chicken egg inoculation confirmed infectivity in test mallards three days after experimental infection with the virus. Furthermore, the virus was no longer viable in the fecal samples following irradiation.

### Bioassay

All six trained mice demonstrated greater than 80% concordance (choosing the maze arm associated with fecal odors from infected ducks) in 13 training sessions. These mice generalized the trained response correctly for all within-duck comparisons (pre- vs. post-infection for ducks #5, 11, and 12; Table 3). As anticipated, trained mice did not discriminate between pre- and post-infection samples from sham-treated ducks (#1 and 2; Table 3). Together, these results indicate that trained mice associated the water reward with the odor of H5N2 infection (not individual duck identity). Furthermore, lack of discrimination among feces from non-infected ducks (Ducks #1 and 2; Table 3) during generalization sessions indicated that sample collection period (pre- vs. post-infection) was not the source of within-duck fecal odor changes.

**Table 3:** Bioassay results of unrewarded generalization trials (pair-wise, within-subjects comparisons of feces collected before and after experimental AIV infection).

Duck	Treatment	% Correct	*z*	*p* *	Sessions	Trials
1	Control	57%^1^	1.03	0.152	2	56
2	Control	56%^1^	0.385	0.350	1	27
5	H5N2	84%	4.67	<0.0001	2	50
11	H5N2	72%	3.20	0.0007	1	28
12	H5N2	72%	2.00	0.0228	1	25

In all cases, mice were trained to select the maze arm associated with the odor of feces collected after experimental infection with avian influenza.

* Binomial test probability with null hypothesis % Correct = 50%.

1 Indicates rate of selection of post-sham infection feces sample.

Results from between-duck generalizations were not as definitive. As would be predicted from mice trained to identify the odor of feces from AIV-infected ducks, mice did not discriminate between feces samples collected from two ducks collected during the pre-infection period (duck 12 versus duck 1; Table 4). However, trained mice were also expected to select the maze arm scented with the odor of infected feces when paired with feces of healthy ducks, even feces collected in the post-infection period. Although, trained mice correctly identified post-infection feces (duck 5) when paired with the post-infection feces from control duck 2 (Table 4), the mice did not discriminate when duck 5 post-infection feces was paired with post-infection feces from control duck 1 (Table 4).

**Table 4:** Bioassay results of unrewarded generalization trials (pair-wise, between-subjects comparisons of feces).

Comparison		% Correct	*z*	*p*	Sessions	Trials
1 - Pre	12 - Pre	42%^1^	0.588	0.278	1	26
2 - Post	5 - Post	71%^2^	3.074	0.0011	2	56
1 - Post	5 - Post	45%^2^	0.858	0.195	3	87

In all cases, mice were trained to select the maze arm associated with the odor of feces collected after experimental AIV infection. “Pre” indicates feces collected prior to experimental infection; “Post” indicates collection after infection. Ducks 5 and 12 were experimentally infected with avian influenza. Ducks 1 and 2 received a sham treatment. Responses were subjected to tests of binomial proportions equal to 50%.

1 Indicates rate of selection of 12 - Pre sample.

2 Indicates rate of selection of 5 - Post sample.

### Chemical Analysis

Data processing of headspace data identified >1900 significant mass spectral responses for each sample. Reconstruction of a composite chromatogram from these responses indicated that 96 individual chromatographic peaks were present in the headspace of the fecal samples. Principal Components Analysis (PCA) of headspace data identified two mass spectral responses that adequately segregated feces samples on the basis of AIV infection status. These responses were tentatively identified as acetoin (m/z = 88 at scan 2141) and 1-octen-3-ol (m/z = 57 at scan 2876).

Peak area ratios (1-octen-3-ol:acetoin) obtained from solvent extraction ranged from 0 to 0.88 across all ducks and collection periods (Table 5). Feces from infected ducks yielded significantly lower peak area ratios (mean = 0.089) than feces from non-infected ducks (mean = 0.535; p = 0.0014 Table 5). Though not highly significant themselves, the low peak ratios of infected ducks were a product of increased acetoin peak responses (p = 0.095) and reduced 1-octen-3-ol responses (p = 0.10) resulting from AIV infection. Results of method evaluation experiments indicated the method was highly repeatable (5.8%) and clean-up of the solvent extracts did not impact the peak ratio response (p = 0.32).

**Table 5:** Peak area responses (×10^5^) of acetoin and 1-octen-3-ol determined in ethanol extracts of duck feces.

		Non-infected Duck Samples				Infected Duck Samples			
Duck	Treatment	Period	Acetoin	1-Octen -3-ol	Ratio	Period	Acetoin	1-Octen -3-ol	Ratio
1	Control	Pre	71	31.1	0.44				
2	Control	Pre	159	33.2	0.21				
1	Control	Post	29	25.6	0.87				
2	Control	Post	47	29.5	0.62				
3	H5N2	Pre	77	44.6	0.58	Post	476	34.9	0.07
4	H5N2	Pre	63	51.6	0.81	Post	179	24.3	0.14
5	H5N2	Pre	47	41.2	0.88	Post	109	21.0	0.19
12	H5N2	Pre	103	20.1	0.19	Post	646	21.3	0.03
13	H5N2	Pre	92	18.8	0.20	Post	1307	13.2	0.01
Mean			76.5	32.9	0.535		543	22.9	0.089
S.D.			38.5	11.1	0.29		480	7.9	0.076
p-value							0.095	0.10	0.0014

Responses of individual compounds are normalized for sample mass. Peak ratio equals 1-octen-3-ol response divided by acetoin response and is thus independent of sample mass. P-values correspond to differences of non-infected and infected ducks for the three different responses.

## Discussion

Trained mice readily discriminated feces of non-infected and infected mallards on the basis of volatile metabolites during training and generalized this response to novel fecal samples differing in infection status (Table 3). That a distinctive fecal odor difference was recognized by trained mice suggests that AIV infection in mallards may be “advertised” to other members of the population as a change in fecal odor. However, not all between-subjects comparisons were successfully discriminated by trained mice (Table 4). Significant individual odor variation among ducks may have made some between-subjects discriminations difficult for trained mice. Thus, it is probable that the odor cues used for discrimination are present both before and after AIV infection, except that infection produces a significant quantitative change in these volatiles. Chemical analyses similarly indicated that fecal volatiles changed quantitatively due to infection. The significant decreases in peak area ratios suggest that decreased 1-octen-3-ol and increased acetoin peak responses are associated with AIV infection.

These compounds (1-octen-3-ol and acetoin) have been identified as potential biomarkers for diagnosing gastrointestinal diseases in humans [27]. Acetoin is an enzymatic decarboxylation product of pyruvate. Many *Bacillus* bacteria, which produce acetoin from pyruvate in the presence of glucose [28], have been identified in duck feces [29]. Additionally, accumulation of cellular pyruvate resulting from viral infection has been known for some time [30]. Multiple type A influenza variants have been shown to impact intracellular glycolytic flux resulting in increased production of pyruvate [31]. Thus, elevated levels of acetoin observed in feces of infected mallards may have resulted from increased pyruvate available to gastrointestinal bacteria. As an enzymatic product, it is unlikely that fecal acetoin was an artifact of sample irradiation.

Two test systems (i.e. trained biosensors and chromatographic analysis) independently confirmed that alterations of fecal volatiles corresponded with AIV infection. However, there is no evidence that acetoin and 1-octen-3-ol represent the very same cues learned by the biosensors when they were trained to discriminate feces on the basis of infection status. Fecal odors associated with infection may involve many aspects of disease pathology and likely result in a myriad of qualitative and quantitative alterations. It is also unclear whether other infectious agents may produce similar or different changes in fecal volatiles. However, pathogen specificity is not a prerequisite for communicating health status to conspecifics. Odor changes in response to infection may be as general as fluctuation of body temperature, yet detectible changes could alert members of the brood to the presence of a potentially transmissible pathogen.

Chemical communication of infection can be adaptive to either the host population (causing individuals in the brood to avoid infected conspecifics) or the pathogen (making infected individuals attractive to other conspecifics). Avoidance of urine odors associated with influenza has been demonstrated in mice [10]. A number of studies have documented that parasite infection alters body odor and the odor of infected mammals is avoided [32], [33]. Conversely, children harboring the parasite *Plasmodium falciparum* attracted twice as many mosquitoes as children uninfected with the malarial parasites [14]. This conflict (attractiveness vs. avoidance) represents a fascinating aspect of pathogen recognition. The host and pathogen genomes can be thought as engaging in a struggle: the pathogen benefits when infected individuals attract new hosts – while healthy individuals benefit by avoiding infected individuals. While either outcome is possible, the chemical means for communicating infection exists. In the case of AIV infection in mallards, the virus and native gut bacteria appear to work in concert to signal the presence of an exogenous pathogen via alteration of individual fecal odor. Furthermore, recognition of these signals by brood mates may be linked to social interactions and has the potential to influence pathogen transmission.
